# The Editor Was Tired

**Published:** 1901-02

**Authors:** 


### Editorial.

### THE EDITOR WAS TIRED.

In the announcement of the suspension of the *Indiana Dental Journal* the editor gives the above pathetic reason for closing up the work to which his life had been devoted for the past three years. He characteristically remarks, "We announce the suspension of publication of the *Indiana Dental Journal* with the close of Volume III. . . . Further publication is suspended because the editor is tired. . . . It was conceived, started, paid for, conducted, and edited by two dentists for the first two years. No other persons, no firm, corporation, association, municipality, or government ever had any interest, real or otherwise, in its publication. . . . But the grind of it! Some of those thirty-six numbers were put together on the train. Editorials were written in various hotels at numerous places. Sundays, that should have been devoted to pious meditation, were sacrificed to editorial work, and evenings that might have been profitably used for sandpapering the intellect were immolated on the altar of original communications."

This somewhat humorous valedictory contains so much of truth that the serious side of it will appeal to every editor. The eternal grind is forcibly and truthfully described, and has been the experience of every conductor of a journal.

It is with more than the usual feelings of regret that this, one of the best on the list of exchanges, has been forced to the wall. The editor was no doubt tired, but if we may be permitted to read between the lines, there was a deeper reason underlying this sacrifice, and the editor, if so disposed, could have added something to our knowledge of journal publication had he given his readers the facts.

It is no new thing in the experience with professional journalism, that ventures are started and speedily find an early grave, and while this is being written the oldest dental journal is understood to have succumbed to the tide that waits for no man or journal, but engulfs many sooner or later, unless governed by that propelling and supporting force, capital, to which must be superadded an energy that will overcome all resistant obstacles.

It is not always true that the fittest survive. The weaklings are many, and, like their human prototypes, they pass through the diseases of childhood, and, while they may never reach a maturity of strength, manage somehow to live.

The journalistic craze seems to attack individuals and societies periodically. Exactly why this almost overpowering idea possesses some it is difficult to tell. In many instances it is inspired by an ambition to be an editor; in others it is the growth of a feeling that the society or section of country should be represented in the literature of their profession, whatever that may be.

This in one sense is a laudable ambition in that it aims at progress, but unfortunately this is not usually the result, for every new effort of the kind simply dilutes the material within reach, thus lowering the literary standard of every periodical.

The establishment of a journal should be based on an absolute want. It should represent something, or it has no reason for its existence. In dentistry it is not difficult to comprehend why a supply house feels the need of an organ to advertise its wares, and because of this, dental journals have sprung up everywhere, all claiming to represent the dental profession. In order to make this appear they have engaged well-known men to act in the editorial capacity, and then circulating each issue without regard to subscription-lists. That this meets their needs is evident from their growth, but it is absurd to speak of these as dental journals. They represent trade and nothing else, and their use of the dental name would be an imposition upon their readers were it not for the fact that those who take these journals have but one object, that of reading the advertisements. All of these journals must not be classed in the same category. There are three or four that aspire to be something more than mere trade organs and have succeeded in maintaining a reputation based on their dental and literary qualities, but these are exceptional.

If there are any indulging the hope that in the near future they may be able to launch a journal upon the world of dentistry, we would earnestly ask them to consider the wrecks of the past and then submit their plans to some one of experience.

A successful journal is a serious burden even where the finances are most satisfactory. The editor is always tired. The monotonous round of copy, proof, and original editorial work that must be prepared; the labor that is never finished; the months that have passed to be succeeded by other months of toil; and then, the misery of it all, the critical man always regards the editor as a shining mark for his best barbed shaft. Were this always the tired editor's reward, life would be intolerable, but the compensation comes occasionally, very occasionally, when some generous soul will write a pleasant epistle of appreciation of the editor's work.

The truth is, that we have in dentistry too many journals of a certain character, and altogether too few of the recognized organs of the profession. In fact, so far as known, there is now only one in the United States. There was a time when three existed, but necessity, which recognizes no law, has driven two of these into the hands of the dealer in dental wares. The reason for this is that there is no such thing as a true dental profession. Did this exist, there would not be any difficulty in having more than one journal to minister to its needs. The apathy of dentists to the interests of their calling is simply appalling. Were it not for the fact that there is some hope for a better state of things through more enlarged intelligence, those who have struggled to bring about a higher professional life would retire discouraged.

It was a noble effort on the part of the two dentists who kept the *Indiana Dental Journal* alive for two years, and they deserved a better fate; but it is no new experience, and as time passes there will be other sacrificial victims upon the same altar, for human nature is the same at all periods, and the sad experience of one seems only to serve as a stimulus to further efforts to reach the same melancholy goal.

